# Exploratory Biomechanical Comparison of Three Posterior Pelvic Ring Fixation Strategies in a Standardized Tile C1.2 Synthetic Model

**DOI:** 10.3390/diagnostics16091273

**Published:** 2026-04-23

**Authors:** Adrian Claudiu Carp, Awad Dmour, Radu Ștefănoiu, Nicolae Șerban, Mihnea-Theodor Sîrbu, Bogdan Puha, Norin Forna, Liliana Savin, Alexandru Filip, Dragoș-Cristian Popescu, Paul-Dan Sîrbu, Bogdan Veliceasa

**Affiliations:** 1 Grigore T. Popa University of Medicine and Pharmacy, 700115 Iasi, Romania; adrian-claudiu.carp@umfiasi.ro (A.C.C.); mihnea-theodor.sirbu@umfiasi.ro (M.-T.S.); bogdan.puha@umfiasi.ro (B.P.); norin.forna@umfiasi.ro (N.F.); liliana.savin@umfiasi.ro (L.S.); alexandru-filip@umfiasi.ro (A.F.); dragos.popescu@umfiasi.ro (D.-C.P.); paul.sirbu@umfiasi.ro (P.-D.S.); bogdan.veliceasa@umfiasi.ro (B.V.); 2 Department of Orthopedics and Trauma/Surgical Science (II), Faculty of Medicine, Grigore T. Popa University of Medicine and Pharmacy, 16 University Street, 700020 Iasi, Romania; 3 Materials Science and Engineering Faculty, National University of Science and Technology Politehnica Bucharest, 060042 Bucharest, Romania; radu.stefanoiu@upb.ro (R.Ș.); nicolae.serban@upb.ro (N.Ș.)

**Keywords:** pelvic ring injury, posterior fixation, sacroiliac screw fixation, biomechanics, synthetic pelvis, axial stiffness

## Abstract

**Background**: Tile C1.2 pelvic ring injuries are characterized by combined rotational and vertical instability and require reliable posterior stabilization. The aim of this exploratory biomechanical study was to compare the construct-level mechanical behavior of three posterior pelvic ring fixation strategies in a standardized Tile C1.2 injury model while maintaining identical anterior symphyseal fixation in all specimens. **Methods**: Nine fourth-generation composite pelvic specimens with a simulated Tile C1.2 injury pattern were allocated to three groups (*n* = 3 per group) according to posterior fixation method: anterior sacroiliac plating, sacroiliac screw fixation, and ilioiliac plate fixation. All specimens received the same anterior symphyseal plate. Mechanical testing was performed under monotonic axial compression using a universal testing machine and a custom acetabular support designed to ensure reproducible load transmission. A preload of 50 N was applied before data acquisition, after which displacement was zeroed. Loading was then continued up to a predefined maximum load of 1.9 kN. Axial displacement was obtained from actuator travel, and apparent axial secant stiffness was evaluated at predefined load levels. **Results**: Across the tested loading range, sacroiliac screw fixation demonstrated the lowest axial displacement and the highest apparent axial secant stiffness, whereas ilioiliac plate fixation showed the greatest displacement and the lowest stiffness values. Anterior sacroiliac plate fixation showed intermediate mechanical behavior. No structural failure occurred within the tested load range. **Conclusions**: Within the limits of this small synthetic biomechanical study, the investigated posterior fixation strategies showed different construct-level displacement and stiffness profiles under monotonic axial compression when anterior fixation was kept constant. Among the tested posterior constructs, sacroiliac screw fixation was associated with lower displacement and higher apparent stiffness within this experimental model.

## 1. Introduction

Pelvic ring fractures are severe traumatic injuries associated with substantial morbidity and mortality, particularly when instability involves both the anterior and posterior components of the ring [1,2,3]. Among these injuries, Tile type C fractures are characterized by complete disruption of pelvic ring stability. Within this category, Tile C1.2 injuries represent unilateral pelvic ring injuries involving disruption of both the anterior and posterior ring, resulting in combined rotational and vertical instability. Clinically, these injuries are associated with marked instability, impaired load transfer, and a frequent need for operative stabilization of the posterior pelvic ring. Effective stabilization is essential to restore load transfer between the axial skeleton and lower extremities and to reduce the risk of secondary displacement during mobilization [4,5].

The posterior pelvic ring plays a dominant role in overall pelvic stability, and restoration of posterior ring integrity is widely regarded as a priority in the management of unstable pelvic fractures [3,6]. Sacroiliac screw fixation has become a commonly used method of posterior stabilization because of its minimally invasive character and favorable biomechanical profile. However, a range of posterior fixation strategies remains in clinical use, and construct behavior may vary according to implant configuration, fixation pathway, and loading conditions [4,6,7].

Anterior pelvic ring stabilization, most commonly achieved through symphyseal plating, is frequently performed in unstable pelvic ring injuries. Nevertheless, the mechanical interaction between anterior and posterior fixation components remains incompletely defined, particularly under axial loading conditions and across different experimental models [8,9,10]. Existing biomechanical studies have provided valuable insights into pelvic fixation strategies, but the published literature remains heterogeneous with respect to injury patterns, fixation constructs, specimen type, and loading protocols, which complicates direct comparison between studies [4,11,12,13,14]. In addition, relatively few investigations have focused specifically on standardized Tile C1.2 injury models, and recent computational work has further suggested that fixation strategy may influence displacement behavior and stress distribution in these fracture patterns [15].

Accordingly, the aim of the present exploratory descriptive biomechanical study was to compare the construct-level mechanical behavior of three posterior pelvic ring fixation strategies in a standardized Tile C1.2 fracture model using fourth-generation composite pelvic specimens while maintaining identical anterior symphyseal fixation in all specimens. The novelty of the present study lies in the controlled biomechanical comparison of three posterior fixation strategies in a standardized Tile C1.2 synthetic model while maintaining an identical anterior fixation construct in all specimens. By keeping the anterior construct constant, the study was designed to isolate the posterior fixation method as the principal biomechanical variable under the defined loading conditions. It was hypothesized that sacroiliac screw fixation would demonstrate higher construct stiffness and lower displacement than anterior sacroiliac plating and ilioiliac plate fixation within the tested experimental configuration [15].

## 2. Materials and Methods

### 2.1. Study Design

This study represents a controlled experimental biomechanical investigation designed to compare the construct-level mechanical behavior of three different posterior pelvic ring fixation strategies in a standardized Tile C1.2 pelvic fracture model. The anterior fixation construct was kept constant across all specimens, while the posterior fixation method constituted the independent variable. The primary outcome measures were axial displacement and apparent axial secant stiffness under monotonic compression.

Nine composite pelvic specimens were used and allocated into three experimental groups, with three specimens per group. Each specimen was tested only once in order to avoid cumulative material deformation and repeated-loading bias. The sample size of three specimens per group was selected in accordance with the exploratory nature of this controlled biomechanical laboratory investigation and the use of highly standardized composite pelvic specimens intended to minimize inter-specimen variability. The primary objective was to characterize relative construct behavior under identical experimental conditions rather than to establish definitive population-level estimates.

All biomechanical testing procedures were conducted at the Materials Science and Engineering Faculty, National University of Science and Technology Politehnica Bucharest, Bucharest, Romania. The experimental setup, custom testing jigs, and mechanical testing protocol were developed and implemented within the facilities of the host institution, ensuring controlled laboratory conditions and reproducible boundary constraints throughout the study.

This experimental biomechanical investigation did not involve human participants or clinical observational data; therefore, reporting guidelines such as STROBE were not applicable. The study should be regarded as a Level V experimental biomechanical investigation.

### 2.2. Specimens and Composite Pelvic Model

Biomechanical testing was performed using standardized composite pelvic models (Pelvis with partial fusion, Absolute 4th generation, 17 PCF solid foam core, large size, SKU 3415-1, Sawbones, Pacific Research Laboratories, Vashon, WA, USA). These models consist of a short fiber-reinforced epoxy cortical shell and a solid polyurethane cancellous core, providing consistent geometry and mechanical properties with minimal variability between specimens.

The selected model presents a fused pubic symphysis and a fused right sacroiliac joint, while the left sacroiliac joint is not connected by design. This configuration was intentionally used to reproduce a complete unilateral pelvic ring disruption. Composite specimens were selected in order to ensure high reproducibility of fixation performance and to minimize the confounding variability associated with cadaveric bone quality [12,13].

### 2.3. Fracture Simulation

A Tile C1.2 pelvic injury pattern was simulated in all specimens. The left sacroiliac joint was already disconnected by the manufacturer, reproducing posterior ring instability. To complete the anterior ring disruption, the fused pubic symphysis was sectioned using a circular oscillating saw, thereby creating a complete symphyseal dissociation.

This procedure resulted in a unilateral disruption of both the anterior and posterior pelvic ring, producing a vertically and rotationally unstable hemipelvis, consistent with the biomechanical definition of Tile type C pelvic injuries. All osteotomies were performed using the same technique and at the same location to ensure uniformity across specimens. No ligamentous structures were simulated, which represents an inherent limitation of the composite pelvic model and was considered when interpreting the biomechanical findings.

### 2.4. Fixation Techniques and Implant Configuration

All specimens underwent anterior and posterior stabilization. The anterior fixation construct was identical in all specimens, whereas the posterior fixation method differed between the three groups.

Anterior stabilization was performed in all specimens using a contoured 3.5 mm stainless steel reconstruction plate with 7 holes applied across the pubic symphysis. Fixation was achieved with 6 cortical screws. Screw length was selected according to local specimen geometry in order to achieve stable fixation while avoiding implant overlap. Manual screw tightening was performed by the same surgeon in all specimens in order to reduce technical variability.

Three posterior fixation constructs were evaluated, each applied to three specimens.

#### 2.4.1. Group A: Anterior Sacroiliac Plate Fixation

**Figure 1:**
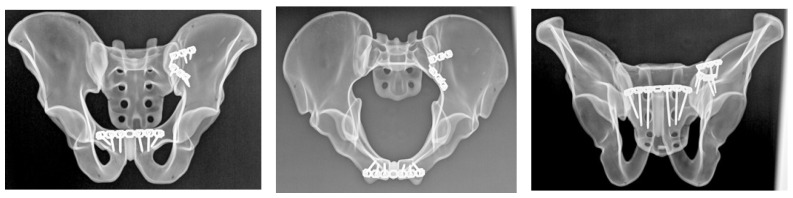
Anteroposterior, inlet, and outlet radiographs demonstrating anterior sacroiliac plate fixation of the posterior pelvic ring in the Tile C1.2 fracture model.

Anterior sacroiliac stabilization was performed using two contoured stainless steel reconstruction plates, each with 3 holes, spanning the sacroiliac joint in a V-shaped configuration (Figure 1). Fixation was achieved with one 3.5 mm stainless steel cortical screw into the sacral ala and two 3.5 mm bicortical screws into the ilium for each plate. Screw lengths were selected according to plate size and local bone geometry in order to achieve stable fixation without implant overlap.

#### 2.4.2. Group B: Sacroiliac Screw Fixation

**Figure 2:**
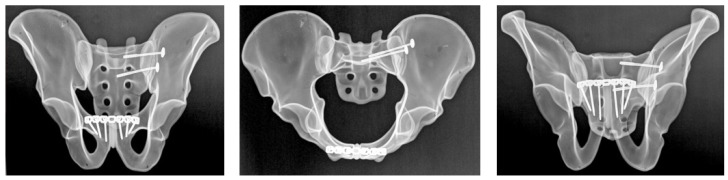
Anteroposterior, inlet, and outlet radiographs showing dual-level sacroiliac screw fixation at the S1 and S2 levels for posterior pelvic ring stabilization.

Posterior pelvic ring stabilization was achieved using two cannulated, partially threaded sacroiliac screws with a diameter of 6.5 mm and a thread length of 16 mm, inserted across the left sacroiliac joint at the S1 and S2 levels (Figure 2). Washers were used in all cases to optimize load distribution at the entry point and to reduce the risk of screw head penetration into the bone during tightening. Screw trajectory and screw length were selected according to previously described anatomical corridors and biomechanical principles in order to ensure adequate purchase within the sacral body [16]. After insertion, all specimens underwent radiographic verification to confirm appropriate screw position and trajectory.

#### 2.4.3. Group C: Ilioiliac Plate Fixation

**Figure 3:**
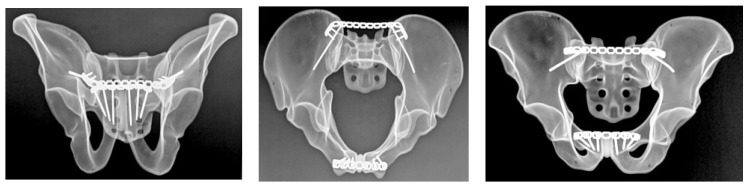
Anteroposterior, inlet, and outlet radiographs illustrating ilioiliac plate fixation spanning the posterior pelvic ring.

Posterior stabilization in this group was achieved using a 3.5 mm stainless steel reconstruction plate with 14 holes spanning the posterior pelvic ring (Figure 3). Fixation was performed with 6 cortical screws, with 3 screws placed on each side. Plate contouring and screw placement followed standard posterior pelvic ring fixation principles. Screw length was selected according to local specimen geometry in order to achieve stable fixation while avoiding implant overlap.

All implants used in this study were stainless steel, reflecting routine clinical practice in pelvic trauma surgery. All fixation procedures were performed by the same surgeon to reduce inter-operator variability.

After fixation, all specimens underwent radiographic verification using standard pelvic radiographs obtained in three projections: anteroposterior and two oblique cranial-caudal projections corresponding to inlet- and outlet-type views. These radiographs were used to confirm correct implant positioning, intraosseous screw placement, and absence of obvious cortical breach. They were not used to assess fracture reduction or alignment.

### 2.5. Experimental Test Setup

Each specimen was mounted in a custom-designed testing jig developed to reproduce controlled axial loading under standardized boundary conditions. The specimen was supported inferiorly by two cylindrical metallic columns positioned beneath the ischial regions, allowing vertical load transfer while maintaining pelvic alignment.

Compressive load was applied through a custom tubular acetabular support, with the external diameter matched to the internal acetabular geometry, in order to ensure uniform force transmission and to avoid stress concentration at the load application point.

To reduce parasitic rotational motion during compression, the pelvis was additionally stabilized at the level of the anterior superior iliac spines using two metallic fixation elements connected to the baseplate by adjustable tensioned links. This configuration limited rotational degrees of freedom while allowing for controlled vertical displacement of the construct, thereby ensuring that the recorded displacement reflected construct-level response within the experimental setup rather than gross specimen motion.

**Figure 4:**
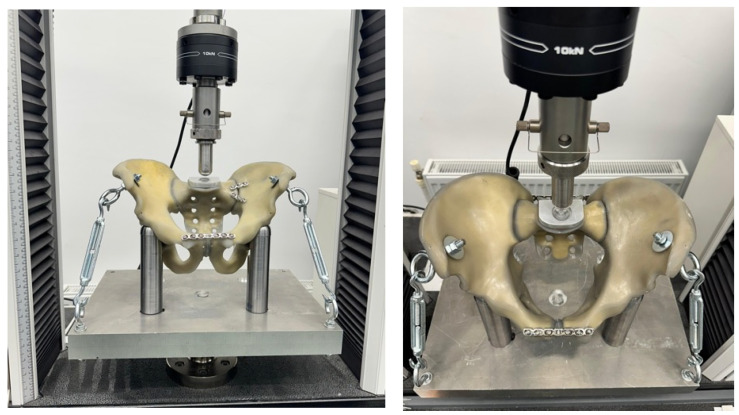
Experimental test setup depicting specimen positioning, boundary conditions, and axial load application through a custom acetabular support.

The design of the jig allowed for reproducible positioning of all specimens and ensured consistent boundary conditions throughout testing (Figure 4).

### 2.6. Mechanical Testing Protocol

All specimens were tested using a universal mechanical testing machine (Instron 3382, Instron Corporation, Norwood, MA, USA) equipped with a 10 kN load cell. Testing was performed under monotonic compressive loading at a displacement rate of 1 mm/min and at an ambient temperature of 20 °C.

A preload of 50 N was first applied to ensure full seating of the construct, after which actuator displacement was zeroed. Loading was then continued under monotonic axial compression up to a predefined maximum load of 1.9 kN. This limit was selected as a subfailure but physiologically relevant axial compression magnitude, intended to approximate the order of loading transmitted through the hip–pelvic complex during weight-bearing while avoiding catastrophic destruction of the composite specimens [3,17]. No specimen reached structural failure within the tested load range.

Axial displacement was obtained from actuator (crosshead) travel recorded by the testing machine. Accordingly, the recorded displacement reflects global system deformation rather than isolated local interfragmentary motion.

Force and displacement data were recorded continuously throughout the test for subsequent analysis.

### 2.7. Cyclic Loading Consideration

Cyclic loading was not performed in the present study. The primary objective was to evaluate construct-level mechanical behavior under monotonic axial compression in a standardized experimental model. Fatigue testing was intentionally excluded in order to reduce methodological complexity and to allow for direct comparison between fixation constructs under identical boundary conditions.

### 2.8. Outcome Measures and Data Analysis

Biomechanical stability was assessed using load–displacement behavior and apparent axial secant stiffness. Because displacement was zeroed at preload, apparent axial secant stiffness was calculated at 200, 400, 600, 800, and 1000 N according to the formula k = (F − 50)/x_F, where F represents the applied load level, 50 N is the initial preload, and x_F is the corresponding displacement recorded at that load. Secant stiffness was chosen to enable standardized comparison at predefined load levels without assuming a fully linear load–displacement response. Given the limited number of specimens in each group, the present biomechanical investigation was designed as a descriptive study. Descriptive mean values were calculated for each fixation construct across the tested loading range. No inferential statistical testing was performed, and the findings should therefore be interpreted as descriptive mechanical trends observed within the experimental model.

## 3. Results

### 3.1. Load–Displacement Behavior

All specimens completed the monotonic axial compression protocol up to the predefined maximum load of 1.9 kN. No construct failure was observed within the tested loading range. No implant breakage, macroscopic fracture of the composite bone, or abrupt loss of load-bearing capacity was identified before the upper load limit was reached.

**Figure 5:**
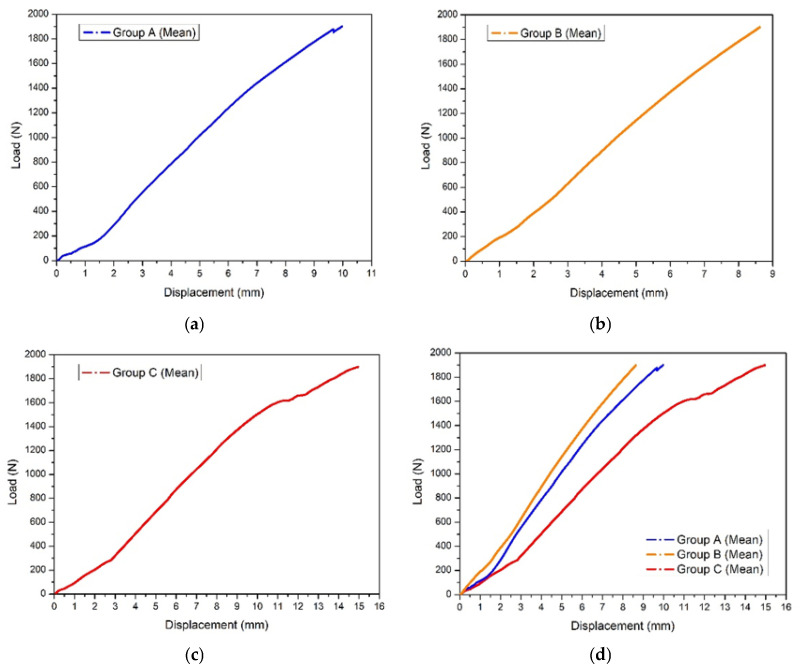
Load–displacement diagrams for the three investigated specimen groups: (**a**) mean curve for Group A; (**b**) mean curve for Group B; (**c**) mean curve for Group C; (**d**) comparative mean curves for all three groups.

Mean load–displacement curves for the three posterior fixation strategies are shown in Figure 5. Across the tested loading range, sacroiliac screw fixation demonstrated the lowest displacement, anterior sacroiliac plate fixation showed intermediate displacement behavior, and ilioiliac plate fixation demonstrated the greatest displacement under equivalent applied loads. The ilioiliac plate construct showed a slightly less linear load–displacement profile at higher load levels, whereas the sacroiliac screw construct showed a more linear load–displacement response across the tested range.

When the mean curves were compared directly, the same ranking was maintained throughout the loading protocol, indicating different construct-level axial displacement profiles across the investigated posterior fixation strategies under the defined experimental conditions.

### 3.2. Apparent Axial Secant Stiffness

**Figure 6:**
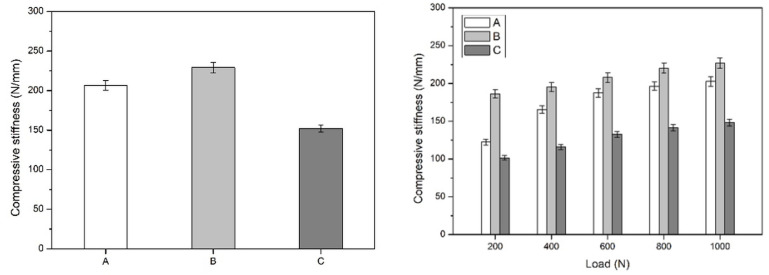
Apparent axial secant stiffness derived from recorded displacement at predefined load levels for the three investigated specimen groups.

Descriptive mean apparent axial secant stiffness values derived from the recorded displacement are summarized in Table 1 and illustrated in Figure 6. Across the evaluated loading range, sacroiliac screw fixation demonstrated the highest stiffness values, anterior sacroiliac plate fixation showed intermediate values, and ilioiliac plate fixation demonstrated the lowest stiffness values. This ranking was maintained at all predefined load levels.

At the lower loading range, the difference between sacroiliac screw fixation and anterior sacroiliac plate fixation was more evident, whereas at higher loads, the stiffness values of these two constructs became more closely aligned. In contrast, ilioiliac plate fixation consistently remained the least stiff construct throughout testing. These findings indicate a consistent descriptive mechanical ranking among the tested posterior fixation strategies when anterior fixation was kept constant.

The presented values should be interpreted as descriptive mean construct-level mechanical observations obtained from three specimens per group; no inferential statistical testing was performed.

### 3.3. Construct Integrity During Testing

Throughout testing, all constructs maintained structural integrity within the applied loading range. No visible implant deformation, composite bone fracture, or abrupt mechanical collapse was observed. Accordingly, the present experiment should be interpreted as a subfailure monotonic axial compression study describing displacement and stiffness behavior rather than as a construct failure study. Given the exploratory design and the limited number of specimens per group, the observed between-group differences should be interpreted as descriptive biomechanical trends within the tested synthetic model and under the defined boundary conditions.

## 4. Discussion

The present exploratory descriptive biomechanical study compared the construct-level mechanical behavior of three posterior fixation strategies in a standardized Tile C1.2 pelvic ring injury model while maintaining identical anterior symphyseal fixation in all specimens. Under the defined experimental conditions, sacroiliac screw fixation was associated with the lowest displacement and the highest apparent axial secant stiffness, whereas anterior sacroiliac plate fixation showed intermediate mechanical behavior and ilioiliac plate fixation showed greater displacement and lower stiffness values. Because all specimens received the same anterior fixation construct, the present study does not permit conclusions regarding the biomechanical advantage of combined fixation over isolated fixation. Rather, it provides a controlled descriptive comparison of posterior fixation strategies under standardized anterior fixation conditions.

The experimental protocol was designed to evaluate construct behavior under monotonic axial compression rather than fatigue performance or ultimate failure strength. Loading was applied up to a predefined maximum load of 1.9 kN, and no structural failure occurred within the tested load range. Accordingly, the present findings should be interpreted as subfailure construct-level mechanical observations describing displacement and stiffness behavior within a constrained synthetic model rather than as definitive evidence of clinical superiority or failure resistance.

### 4.1. Posterior Fixation Strategy and Sacroiliac Screw Configuration

The posterior pelvic ring provides the dominant contribution to overall pelvic stability, and restoration of posterior ring integrity remains a central principle in the management of unstable pelvic injuries [3,18,19,20,21]. In the present study, the posterior fixation construct based on dual sacroiliac screws placed at the S1 and S2 levels showed the lowest displacement and the highest apparent axial secant stiffness among the tested configurations. These findings are consistent with previous biomechanical and computational investigations indicating that screw-based posterior stabilization can provide effective resistance to displacement in unstable pelvic ring injuries [4,18,20,21].

In the current experimental model, the use of two 6.5 mm partially threaded sacroiliac screws with washers reflects a commonly used clinical strategy intended to balance fixation strength with procedural safety. Previous biomechanical studies have shown that screw construct behavior is influenced by implant trajectory, bony corridor quality, and the bone–implant interface, particularly at the iliac entry point [4,7,12]. In this context, the observed mechanical performance of the sacroiliac screw group is biomechanically plausible, but should still be interpreted within the limits of the present descriptive design and synthetic test configuration. Because no construct failure occurred during testing, the present study does not provide information regarding the ultimate strength or failure mode of the screw-based construct.

### 4.2. Interpretation of Plate-Based Posterior Constructs

Anterior sacroiliac plate fixation showed intermediate behavior across the tested loading range and remained mechanically closer to sacroiliac screw fixation than ilioiliac plate fixation, particularly at higher loads. In contrast, ilioiliac plate fixation consistently demonstrated the greatest displacement and the lowest apparent axial secant stiffness. These findings indicate that the investigated posterior fixation strategies were associated with different construct-level axial behavior under the defined loading conditions, even when the anterior construct was identical in all groups.

However, these differences should not be overstated. The present study included only three specimens per group and no inferential statistical analysis was performed. Therefore, the observed ranking of fixation constructs should be interpreted as a descriptive mechanical trend rather than as definitive proof that one method is categorically superior to another in all settings. In addition, the present experiment evaluated only monotonic axial compression and did not assess torsional, shear, or cyclic loading, which may affect the relative performance of plate-based and screw-based constructs differently [6,10,18].

### 4.3. Relevance of the Synthetic Model and Boundary Conditions

Fourth-generation composite pelvic models offer important advantages for comparative biomechanical testing, including standardized geometry, reproducible material properties, and reduced inter-specimen variability compared with cadaveric models [7,13,18,22]. For the purpose of the present study, this standardization was useful because it allowed for a controlled comparison of posterior fixation constructs under consistent testing conditions.

At the same time, the experimental model remained only partially physiological. The selected composite pelvis included a fused contralateral sacroiliac joint and a surgically sectioned pubic symphysis, and no ligamentous structures were simulated. Furthermore, rotational freedom was partially constrained through stabilization at the anterior superior iliac spines, and loading was applied through a custom acetabular support designed to ensure reproducible axial force transmission. These design choices increased reproducibility but also reduced the ability of the model to reproduce the full multidirectional biomechanics of an unstable pelvic ring in vivo [12,14,23]. Consequently, the measured displacement and stiffness values should be interpreted as construct-level responses within the boundary conditions of the experimental setup rather than as a complete representation of physiological pelvic mechanics.

### 4.4. Relationship to Previous Computational and Biomechanical Work

The present findings can be discussed alongside prior biomechanical and finite element investigations, but such comparisons should be made cautiously because published studies differ substantially in specimen type, injury model, implant configuration, and loading protocol [4,11,12,13,14,18,22]. The descriptive ranking observed here, with sacroiliac screw fixation showing the lowest displacement and highest apparent stiffness among the tested posterior constructs, is broadly compatible with prior evidence indicating that fixation strategy influences pelvic ring displacement behavior and construct stiffness [4,18,21,22].

At the same time, the current experiment should not be interpreted as validating broader claims regarding combined fixation concepts. Although previous modeling studies have reported altered stress distribution and reduced motion with different combinations of anterior and posterior stabilization [8,10,15,22], the present study did not include an isolated posterior fixation arm or an isolated anterior fixation arm. Accordingly, its contribution is more limited and more precise: it provides a descriptive comparison of three posterior fixation strategies in the setting of identical anterior fixation.

### 4.5. Methodological Considerations and Limitations

Several limitations must be acknowledged when interpreting the present findings. First, the study used only three specimens per group, and no inferential statistical analysis was performed. The reported differences therefore represent descriptive observations rather than definitive quantitative comparisons. Second, displacement was derived from actuator travel rather than from direct local motion tracking at the fracture site. As a result, the reported stiffness values represent apparent construct-level axial stiffness under the defined test conditions rather than direct measurement of local interfragmentary motion. Third, the test protocol was limited to monotonic axial compression and did not include cyclic, torsional, or shear loading. In addition, because cyclic loading was not performed, the present study does not provide information regarding fatigue behavior, progressive loosening, or long-term construct durability. Likewise, the absence of torsional and shear testing limits the interpretation of multidirectional construct stability, which may differ from axial compression behavior alone. Fourth, no construct failure occurred within the applied load range, so the experiment should be regarded as a subfailure mechanical comparison rather than a failure study.

Additional limitations arise from the model itself. Composite pelvic specimens do not reproduce ligamentous tension and viscoelastic soft-tissue contributions to pelvic ring stability, and the fused contralateral sacroiliac joint may alter global ring compliance and load sharing compared with a physiologically mobile pelvis. Radiographic verification was used to confirm implant position, but fracture reduction quality was not quantified, and small differences in reduction may have influenced stiffness and displacement measurements. Taken together, these factors markedly limit external validity and reduce the extent to which the present findings can be translated directly to clinical decision-making.

### 4.6. Clinical and Research Implications

Within these limitations, the present study may still be useful as a controlled biomechanical comparison of posterior fixation constructs in a standardized Tile C1.2 model. The findings suggest that the investigated posterior fixation strategies were associated with different construct-level displacement and stiffness profiles under monotonic axial compression when anterior fixation was kept constant. In this setting, sacroiliac screw fixation showed lower displacement and higher apparent stiffness than the tested plate-based posterior constructs.

Nevertheless, these findings should not be interpreted as evidence that combined fixation is superior to isolated fixation, nor as definitive proof of clinical superiority of one posterior fixation strategy over another. Clinical decision-making must also consider fracture morphology, patient-specific anatomy, bone quality, surgical invasiveness, and implant availability [21,24]. In this context, sacroiliac screw fixation may be clinically attractive because of its minimally invasive character and reduced soft-tissue exposure, whereas plate-based fixation may require wider operative exposure and greater dissection; operative time and technical demands may also differ between techniques, and these factors should be weighed together with the biomechanical findings and potential complication profile [21]. Future investigations should incorporate more physiological models, larger sample sizes, direct local motion tracking, and cyclic and multidirectional loading protocols in order to better define the relative biomechanical roles of different fixation strategies in unstable pelvic ring injuries.

## 5. Conclusions

In this small descriptive biomechanical study, the investigated posterior fixation strategies showed different construct-level displacement and apparent axial secant stiffness profiles in a standardized Tile C1.2 synthetic pelvic model under monotonic axial compression. Among the tested posterior constructs, sacroiliac screw fixation was associated with lower displacement and higher apparent stiffness. These findings should be interpreted as descriptive subfailure biomechanical trends within a constrained experimental model and not as definitive evidence of clinical superiority.

## Figures and Tables

**Table 1:** Descriptive mean apparent axial secant stiffness values for each fixation configuration at the predefined load levels (N/mm).

Group	200 N	400 N	600 N	800 N	1000 N
A (anterior sacroiliac plate fixation)	122.39	165.68	187.57	196.58	202.59
B (sacroiliac screw fixation)	186.27	195.35	207.85	220.37	227.07
C (ilioiliac plate fixation)	101.56	116.14	132.60	141.73	148.21

## Data Availability

The original contributions presented in this study are included in the article. Further inquiries can be directed to the corresponding author.

## References

[1] Garcia M., Firek M., Zakhary B., Brenner M., Hildebrand F., Coimbra R. (2020). Severe Pelvic Fracture in the Elderly: High Morbidity, Mortality, and Resource Utilization. Am. Surg..

[2] Baker J.E., Werner N.L., Burlew C.C. (2024). Management of Pelvic Trauma. Surg. Clin. N. Am..

[3] Vleeming A., Schuenke M.D., Masi A.T., Carreiro J.E., Danneels L., Willard F.H. (2012). The Sacroiliac Joint: An Overview of Its Anatomy, Function and Potential Clinical Implications. J. Anat..

[4] Cardwell M.C., Meinerz C.M., Martin J.M., Beck C.J., Wang M., Schmeling G.J. (2021). Systematic Review of Sacroiliac Joint Motion and the Effect of Screw Fixation. Clin. Biomech..

[5] Morris S.A.C., Loveridge J., Smart D.K.A., Ward A.J., Chesser T.J.S. (2012). Is Fixation Failure After Plate Fixation of the Symphysis Pubis Clinically Important?. Clin. Orthop. Relat. Res..

[6] Tripathi S., Nishida N., Soehnlen S., Kelkar A., Kumaran Y., Seki T., Sakai T., Goel V.K. (2024). Pelvic Ring Fractures: A Biomechanical Comparison of Sacral and Lumbopelvic Fixation Techniques. Bioengineering.

[7] Crist B.D., Pfeiffer F.M., Khazzam M.S., Kueny R.A., Della Rocca G.J., Carson W.L. (2019). Biomechanical Evaluation of Location and Mode of Failure in Three Screw Fixations for a Comminuted Transforaminal Sacral Fracture Model. J. Orthop. Transl..

[8] Song Y., Shao C., Yang X., Lin F. (2022). Biomechanical Study of Anterior and Posterior Pelvic Rings Using Pedicle Screw Fixation for Tile C1 Pelvic Fractures: Finite Element Analysis. PLoS ONE.

[9] Cavalcanti Kußmaul A., Greiner A., Kammerlander C., Zeckey C., Woiczinski M., Thorwächter C., Gennen C., Kleber C., Böcker W., Becker C.A. (2020). Biomechanical Comparison of Minimally Invasive Treatment Options for Type C Unstable Fractures of the Pelvic Ring. Orthop. Traumatol. Surg. Res..

[10] Liu L., Zeng D., Fan S., Peng Y., Song H., Jin D., Zeng L. (2021). Biomechanical Study of Tile C3 Pelvic Fracture Fixation Using an Anterior Internal System Combined with Sacroiliac Screws. J. Orthop. Surg. Res..

[11] Jordan M.C., Bröer D., Fischer C., Heilig P., Gilbert F., Hölscher-Doht S., Kalogirou C., Popp K., Grunz J.-P., Huflage H. (2022). Development and Preclinical Evaluation of a Cable-Clamp Fixation Device for a Disrupted Pubic Symphysis. Commun. Med..

[12] Chatain G.P., Oldham A., Uribe J., Duhon B., Gardner M.J., Witt J.-P., Yerby S., Kelly B.P. (2023). Biomechanics of Sacroiliac Joint Fixation Using Lag Screws: A Cadaveric Study. J. Orthop. Surg. Res..

[13] Girardi B.L., Attia T., Backstein D., Safir O., Willett T.L., Kuzyk P.R.T. (2016). Biomechanical Comparison of the Human Cadaveric Pelvis with a Fourth Generation Composite Model. J. Biomech..

[14] Rudy R.F., Sawa A.G.U., McBryan S., Mugge L.A., Thielen K., Assefa T.G., Lindsey D.P., Polly D.W., Uribe J.S., Kelly B.P. (2025). Impact of Multipoint Pelvic Fixation and Multirod Distal Constructs on Proximal Junction Biomechanics in Cadaveric Specimens. J. Neurosurg. Spine.

[15] Carp A.C., Veliceasa B., Awad D., Filip A., Perțea M., Forna N., Puha B., Tîrnovanu Ș.D., Sîrbu M.T., Pavăl S.D. (2026). Biomechanical Comparison of Three Fixation Constructs for Tile Type C1.2 Pelvic Ring Fractures: A Finite Element Analysis. Life.

[16] Ramadanov N., Zabler S. (2025). Ramadanov–Zabler Safe Zone for Sacroiliac Screw Placement: A CT-Based Computational Pilot Study. J. Clin. Med..

[17] Bergmann G., Deuretzbacher G., Heller M., Graichen F., Rohlmann A., Strauss J., Duda G.N. (2001). Hip Contact Forces and Gait Patterns from Routine Activities. J. Biomech..

[18] Hu P., Wu T., Wang H., Qi X., Yao J., Cheng X., Chen W., Zhang Y. (2019). Biomechanical Comparison of Three Internal Fixation Techniques for Stabilizing Posterior Pelvic Ring Disruption: A 3D Finite Element Analysis. Orthop. Surg..

[19] Wu T., Chen W., Zhang Q., Zheng Z.-L., Lyu H.-Z., Cui Y.-W., Cheng X.-D., Zhang Y.-Z., Yang Y.-J. (2015). Biomechanical Comparison of Two Kinds of Internal Fixation in a Type C Zone II Pelvic Fracture Model. Chin. Med. J. (Engl. Ed.).

[20] van Zwienen C.M.A., van den Bosch E.W., Snijders C.J., Kleinrensink G.J., van Vugt A.B. (2004). Biomechanical Comparison of Sacroiliac Screw Techniques for Unstable Pelvic Ring Fractures. J. Orthop. Trauma.

[21] Kim C.-H., Kim J.W. (2020). Plate versus Sacroiliac Screw Fixation for Treating Posterior Pelvic Ring Fracture: A Systematic Review and Meta-Analysis. Injury.

[22] Lipphaus A., Klimek M., Witzel U. (2021). Comparative Finite Element Analysis of Fixation Techniques for APC II Open-Book Injuries of the Pelvis. Biomechanics.

[23] Dmour A., Toma Ș.-L., Cazac A.-M., Tirnovanu S.D., Dima N., Dmour B.-A., Popescu D.C., Alexa O. (2024). Comparative Biomechanical Analysis of Kirschner Wire Fixation in Dorsally Displaced Distal Radius Fractures. Life.

[24] Tile M. (1988). Pelvic Ring Fractures: Should They Be Fixed?. J. Bone Jt. Surg. Br..

